# Can working with the private for-profit sector improve utilization of quality health services by the poor? A systematic review of the literature

**DOI:** 10.1186/1475-9276-6-17

**Published:** 2007-11-07

**Authors:** Edith Patouillard, Catherine A Goodman, Kara G Hanson, Anne J Mills

**Affiliations:** 1 Health Economics and Financing Programme, London School of Hygiene and Tropical Medicine, London, UK; 2 Kenya Medical Research Institute/Wellcome Trust Research Programme, Nairobi, Kenya

## Abstract

**Background:**

There has been a growing interest in the role of the private for-profit sector in health service provision in low- and middle-income countries. The private sector represents an important source of care for all socioeconomic groups, including the poorest and substantial concerns have been raised about the quality of care it provides. Interventions have been developed to address these technical failures and simultaneously take advantage of the potential for involving private providers to achieve public health goals. Limited information is available on the extent to which these interventions have successfully expanded access to quality health services for poor and disadvantaged populations. This paper addresses this knowledge gap by presenting the results of a systematic literature review on the effectiveness of working with private for-profit providers to reach the poor.

**Methods:**

The search topic of the systematic literature review was the effectiveness of interventions working with the private for-profit sector to improve utilization of quality health services by the poor. Interventions included social marketing, use of vouchers, pre-packaging of drugs, franchising, training, regulation, accreditation and contracting-out. The search for published literature used a series of electronic databases including PubMed, Popline, HMIC and CabHealth Global Health. The search for grey and unpublished literature used documents available on the World Wide Web. We focused on studies which evaluated the impact of interventions on utilization and/or quality of services and which provided information on the socioeconomic status of the beneficiary populations.

**Results:**

A total of 2483 references were retrieved, of which 52 qualified as impact evaluations. Data were available on the average socioeconomic status of recipient communities for 5 interventions, and on the distribution of benefits across socioeconomic groups for 5 interventions.

**Conclusion:**

Few studies provided evidence on the impact of private sector interventions on quality and/or utilization of care by the poor. It was, however, evident that many interventions have worked successfully in poor communities and positive equity impacts can be inferred from interventions that work with types of providers predominantly used by poor people. Better evidence of the equity impact of interventions working with the private sector is needed for more robust conclusions to be drawn.

## Background

Recent years have seen increasing interest in the role of the private sector in health service provision in low- and middle-income countries. Many countries have a vibrant and growing private sector, which is perceived by some to respond to public sector failures. Private providers are argued to deliver services which are more accessible, affordable (particularly where public providers charge official or unofficial user fees) and responsive to the needs and preferences of users [1].

The private sector is also an important source of care for poor and disadvantaged groups within low- and middle-income countries. For instance, in Guatemala, 40 to 45% of the population in the two lower income quintiles sought care in the private sector and in South Africa over 33% of each of the three lower quintiles did so [2]. Similarly, in Nepal, the private sector provided care to more than a third of the lowest income quartile [3]. A review of Demographic Health Survey (DHS) data from 38 countries found high levels of private sector use by those in the lowest socioeconomic quintile for childhood diarrhea (34–96% of children) and acute respiratory infections (37–99% of children) [4]. In seeking to extend coverage of priority interventions, there are numerous operational advantages to working with pre-existing, self-sustaining outlets that are widely used by target populations. For these reasons the private sector represents an important potential partner in efforts to scale up coverage of effective health interventions among the poor.

However, there is a growing body of evidence that the care provided in the private sector is often of low technical quality [5]. For example, private sector treatment of sexually transmitted infections (STIs) [6], tuberculosis [7] and malaria [8] have been demonstrated to have significant shortcomings in terms of quality of diagnosis and correct prescription of medications. Concern about the potential harm caused by private providers, combined with recognition that public sector technical quality is often also poor, has led to interest in developing interventions which will take advantage of the potential offered by the private sector while at the same time addressing its technical failures. There is now a substantial literature on ways that government and non-governmental organizations (NGOs) can work with private health care providers, including social marketing, regulation, training, franchising, accreditation and contracting-out [1,4,5,8-11].

It has been argued that there is "evidence that effective public-private partnerships can increase access, improve equity, and raise quality of health services"[12] and that governments should "urgently engage with private stakeholders to. .... facilitate increased private sector participation" [12]. However, the case that private sector interventions improve equity has not yet been clearly made [13]. Moreover, one might fear that working with private for-profit providers may disproportionately benefit the wealthier members of the community who can afford their charges, and thus exacerbate inequity [14]. This paper addresses this knowledge gap by presenting the results of a systematic review of the impact of private sector interventions on the poor. Section 1 sets the scope of the literature review, providing definitions of key terms and concepts and describes the review methods. Section 2 presents the findings, which are discussed in the final section.

## Methods

The scope of the review was defined as follows:

### Scope of the review

#### Private sector

The private health sector is commonly defined as all providers outside the public sector. This includes a heterogeneous mix of for-profit and not-for profit providers, with varying degrees of formality and qualifications. Our focus was on interventions that work with existing for-profit providers, both formal and informal. While we recognize the importance of private not-for-profit providers, their characteristics and incentives are likely to differ from those of commercial providers, and they were considered beyond the scope of this review. In practice, boundaries between public and private provision may be blurred; both formal and informal cost recovery schemes operate at public facilities [15] and dual practice, where providers work in both the public and private sectors, is widespread [16]. We have not set out to include these mixes of public and private operations, though it is possible that some private outlets involved in the reviewed interventions are run by public sector staff.

#### Health services and public health products

In this paper, health services refer to all in-patient and ambulatory care services [17], and public health products are "those commodities used for treatment of diseases of public health importance or for the promotion of health, which can be provided at the retail level without a 'service' attached to them" [18]. Examples of the latter include condoms and mosquito nets. We addressed only those services and interventions which fall under the classification of biomedical/allopathic, and excluded interventions to improve the quality of "traditional" care.

#### Outcome Measures

We included studies focusing on the impact on either utilization or quality of care. We focused on utilization because it reflects the degree to which multiple barriers to access (e.g. geographic, financial, social) are overcome. In terms of quality, our focus was on both the technical and perceived dimensions of quality. We considered technical quality to be assessed through observation of provider behaviour and of the physical attributes of the practice [18]. For example, we included studies that evaluated providers' practical performance against training or national guidelines (e.g. proportion of cases correctly managed), and those that assessed the adequacy of the premises for the services and products provided (e.g. range of essential drugs available). We also included studies on the more controversial dimension of quality based on patients' perceptions and considered perceived quality as measured by the level of satisfaction expressed by patients. Our review excluded studies that reported intervention impacts in terms of providers' qualifications and knowledge alone.

#### Private sector interventions

A preliminary review identified eight areas of intervention involving the government or NGOs working with the private for-profit sector: social marketing, use of vouchers, pre-packaging of drugs, franchising, training, regulation, accreditation and contracting-out.

*Social marketing *is the application of the tools and concepts of commercial marketing to social and health problems [19], in order to increase population coverage of effective and affordable interventions [10]. Social marketing interventions may include a combination of promotional activities, branding, labelling, pre-packaging and subsidy of public health products.

A *voucher *is a form of demand-side subsidy that the recipient can use as part or full-payment for a product or service from identified providers. The distribution of vouchers can be targeted, to improve access for an identified population group such as the poorest households or pregnant women. Vouchers can either be competitively redeemed where they are exchangeable at a number of different providers [10], or non-competitive where they are assigned to one particular provider [9].

*Pre-packaging *is a strategy to improve provider and patient adherence to treatment regimens, and involves packaging drugs in pre-defined doses adequate for the targeted population group and length of treatment regimen [20].

A *franchise *is a contractual arrangement between a health service provider and a franchise organisation. It aims to improve access to quality- and price-controlled services. Franchisees are trained in standardized practices for which prices are predefined [21] and benefit from advertising of the logo or franchise name. In return, franchisees may be required to comply with minimum sales volume and quality standards and pay a membership fee to the franchisor [22]. Providers are monitored by the franchise organisation, which in public health is generally a government or donor-sponsored NGO which subsidises the network [22].

*Accreditation *is a strategy to improve and control the quality of services provided at organisational or facility level through oversight by an independent quality control evaluation body which may be the government or an NGO. It may include training providers in standardised practices [1]. While accreditation is similar to franchising, the nature of the relationship between the provider/accreditor is often voluntary, compared with the contractual relationship between the franchisee/franchise organization.

*Training *interventions can take various forms including formal training sessions, vendor-to-vendor education, distribution of guidelines and job-aids. Training is often integrated into other interventions, such as franchising, accreditation and social marketing.

*Regulatory *interventions aim to set up and ensure adequate technical quality of the services provided [18]. They take the form of rules, enforcement systems and sanction mechanisms, and can be applied at the levels of health care provider, organisation or facility. At the provider level regulation may include requirements for pre-service training, continuing education, licensing and certification of providers [23]. At the organisational or facility level, regulation may aim to control the location of facilities, their registration and minimum complement of staff or facilities [18]. In addition, consumer protection legislation may be used to oversee medical practices and influence provider behaviour. Pharmaceutical market regulation aims to limit the availability of harmful drugs and unregistered products, minimize drug misuse, control the sale of specific drugs through prescriptions, and regulate drug manufacture and importation [20].

*Contracting out *is a purchasing mechanism used to acquire specified services of a defined quality at an agreed price from a specific private provider and for a specific period of time. Governments may purchase clinical or non-clinical services from private providers to complement public provision [24].

Where interventions employed a mix of strategies we classified them by the primary or main intervention, based on the emphasis given in the paper or report.

#### Poverty

The main focus of this paper was the extent to which the effects of private sector interventions reach the poor. Poverty is a multidimensional construct, notoriously difficult to measure [25]. Various tools are used to assess the poverty status of population groups, in either absolute or relative terms. For instance, household income or consumption, asset ownership and housing conditions, residence status and the level of education attained, are all relevant dimensions. We included in the review all studies that examined the effects of interventions on the poor as measured by any of these indicators, for example those with low household income, lower levels of education, or those living in rural areas. It is recognised that all of these proxies suffer from imperfections, in terms both of measurement and interpretation [26,27].

To determine whether an intervention reaches the poor we took two approaches. First, interventions were deemed to have reached the poor if they benefited generally poor areas based on the study site information provided in the original papers. This was termed an average SES measure. Secondly, interventions were deemed to have reached the poor if the socioeconomic distribution of benefits favoured the most disadvantaged groups within a given population. This was termed a relative SES measure. The distinction between these two criteria is important, and is linked to the difference between measures of absolute and relative poverty. For instance, an intervention that is implemented among private providers in a predominantly rural district of a poor country may improve the quality of care received by all socioeconomic groups in that area (i.e. may have no effect on the relative distribution of access to quality services). However, because most of the population is disadvantaged, it can be judged to be successful in reaching the poor based on an average SES measure. Alternatively, an intervention that generates a greater improvement in utilization of quality care for those in the lowest income quintile compared with the highest, for example by targeting providers who are preferentially used by the poorest, would be judged to have reached the poor based on a relative SES measure.

### Search methodology

Published, grey and unpublished literature was systematically searched. The search topic was defined as evidence on the effectiveness of different strategies of working with existing private-for-profit providers to improve utilization and quality of health services and public heath products for the poor.

The search for published literature used the electronic databases PubMed, Popline, HMIC and CabHealth Global Health applying both controlled vocabulary and free text key terms, and limiting the review to articles published after 1990, in English and French (Table 1).

**Table 1:** Search strategy: key terms and limits

**Key terms**	**Limits**
Private Sector*	*Geographic*
Delivery of Health Care†	Developing countries
Health Care Sector†	Low income countries
Social marketing*	Middle income countries
Marketing of health services†	*Linguistic*
Voucher‡	English
Vouchers‡	French
Franchising‡	*Publication period*
Franchise‡	01/1990 to 05/2006
Accreditation*	
Accredited‡	
Education, Pharmacy†	
Training‡	
Contracting out‡	
Contract services‡	
Contracting‡	
Facility regulation and control ^†	
Regulation‡	
Drugs, Non-Prescription†	
Antimalarials†	
Drug Labeling†	
Drug Packaging†	

*MeSH and free text

† MeSH only

‡ free text only

^legislation and jurisprudence subheading only

The search for grey and unpublished literature focused on reports and documents available on the World Wide Web. Using key terms in Table 1, we searched the sites of Eldis Gateway to Development Information, USAID PSP-*One*, and World Bank Knowledge Services for Private Sector Development. The bibliographies of selected papers were also checked for additional papers.

When all searches were complete, document titles and available abstracts were read and potential sources selected based on the following inclusion criteria.

We included all impact evaluations of interventions working with private for-profit providers in low and middle-income countries that measured the effect of the intervention on either utilization or quality of services. An impact evaluation was defined as a study documenting changes over time (pre-post), or comparing an intervention area with a control area (controlled), or comparing changes over time in an intervention area with changes over time in a control area (pre-post with control), with or without randomisation. We excluded studies using national survey data on aggregate changes in utilization of a service or a product that could not be related to a specific intervention.

We then identified interventions with average socioeconomic status (SES) information on the recipient population, and those which documented relative effectiveness across SES groups.

## Results

A total of 2483 references were retrieved, from which 52 evaluated interventions working with private for-profit providers were identified. The majority concerned training (26) and social marketing (14), with the remainder evaluating contracting-out (3), franchising (6), regulation (2) and accreditation (1) [see Additional file 1].

### Social marketing, vouchers and pre-packaging of drugs

These interventions were grouped together because of their significant overlap. Fourteen evaluated interventions were identified, eleven relating to social marketing one also including a voucher scheme, and 2 including prepackaged treatments. A further 3 interventions focused purely on provision of vouchers.

Of the social marketing interventions, six involved condoms and/or other family planning commodities, and one each involved oral rehydration therapy (ORT), iron supplements, insecticide treated nets (ITNs), STI treatment and malaria/acute respiratory infection (ARI) treatment. All showed significant increases in utilization of programme commodities and services, though of differing magnitudes across interventions. For example, social marketing increased condom use among women in urban Cameroon from 58% to 76% [28], coverage of iron-folic acid supplementation from 6% to 99% in non-pregnant Filipino women [29], and ITN coverage in rural Tanzanian children under 2 years from 10% to 61% [30].

The contraceptive social marketing programmes were implemented in urban settings and the majority targeted adolescent groups. A number of interventions included peer educators as well as distribution of commodities through retail outlets (Soweto, Horizon Jeunes, Tsa Banana, My Future First).

The social marketing programme for ORT in a rural Kenyan district included sales of flavoured ORT sachets through shops and a mass communication and education campaign [31]. In the Philippines, social marketing of iron and folic acid supplements in rural municipalities combined free distribution of iron-folic acid supplementation targeted to pregnant women through the public health system and sales of iron supplements to non-pregnant women by village health workers (VHWs) and drug outlets, as well as training VHWs and school teachers in counselling and information, education and communication (IEC) [29].

The Kilombero and Ulanga Insecticide-Treated Net project (KINET) in Tanzania distributed branded ITNs and net treatment kits through retail outlets, and included a comprehensive IEC campaign [19,30,32-34]. Vouchers reducing the price of nets at retail shops by 17% were distributed to pregnant women and mothers of under-five children attending public clinics.

Vouchers for free nets and treatment from retail outlets were used alongside measles vaccination in a campaign approach in an urban Zambian district [35]. Two other voucher schemes for sexual and reproductive health care (SRHC) were implemented in Nicaragua. The first targeted adolescent girls at various sites, entitling the recipient to free SRHC at NGO, public and for-profit clinics [36,37]. The second scheme provided free STI care at private for-profit and NGO clinics for sex workers and their clients [38].

Two final interventions combined social marketing with prepackaged treatments – for STI treatment for male urethritis in Uganda [39] and prepackaged treatment for childhood malaria and acute respiratory infections in Nigeria [40].

Of the 14 interventions, data on the average SES of the recipient community was provided for only one, the KINET ITN project. This clearly benefited a generally poor population, with an estimated median household monthly expenditure of $77–96 in 1997 [34]. For 12 interventions, only general information on the urban/rural settings was available, and for 1 intervention there was no information aside from location.

Data on the distribution of benefits across socioeconomic groups was provided for 2 of these interventions. The effect of KINET on the socioeconomic distribution of ITNs was documented in three studies. The first demonstrated positive changes in the ratio of net ownership in the lowest to the highest SES quintiles (the equity ratio) from 0.3 in 1997 to 0.6 in 2000 [33]. The second reported a significantly greater increase (of 51%) in household net ownership among the poorest income quartile in the social marketing area compared to 32% in the control area. Similar information for households in village peripheries (likely to be poorer) were 68% and 27% [19]. The third study assessed the socioeconomic distribution of vouchers and, in contrast to the other two, found that none of the households in the lowest quintile had used a voucher towards the purchase of a net compared to 8% in the highest quintile [32]. This may be due to the low share of vouchers in total net sales, itself reflecting low knowledge among mothers of vouchers, with only 28% of women having ever heard of the voucher scheme [41].

Poorer groups also benefited in relative terms in urban Zambia, where the equity ratio for ITN coverage increased from 0.66 to 1.19, with no statistically significant association between wealth and ownership found post-intervention [35].

### Regulation

Of the two evaluated interventions identified, one concerned banning a drug and its combination products in Nepal and one assessed a regulatory intervention to improve quality of pharmacy services in Lao P.D.R.

The pharmaceutical ban prohibited the export, import, local production, transportation, storage, sale and distribution of Analgin (an analgesic and antipyretic drug) and its combination products [42]. As a result, the proportion of retail outlets with Analgin decreased from 96.5% at baseline to 21.2% five months after the intervention and 0% sixteen months after [42].

The regulatory intervention in Lao P.D.R involved intensive supervision of the quality of pharmacy services, applying sanctions when rules were violated, and providing up-to-date regulatory documents and information about particular areas needing improvements [43]. The study compared districts with intensified regulation with normal, control districts. Whilst it could not be established that the intensive intervention had a greater effect than routine regulation, moderate but significant improvements in quality were observed in all districts, with mean availability of essential materials increasing by 34% and mean order in the pharmacy (including the presence of advertisements, and whether drugs were stored in their original packaging away from sunlight) increasing by 19% [43].

For these two interventions, no SES information was provided about the recipient populations.

### Training

Training was by far the most evaluated intervention, with 26 interventions covering different types of private providers: 4 targeted private doctors, 2 private midwives, 8 private pharmacy workers, 6 drug retailers and 6 a mix of provider types. Training interventions aimed to improve the quality of treatment of a range of different conditions. Seven interventions focused on treatment of childhood illness (use of integrated management of childhood illness (IMCI) guidelines, treatment of ARI or diarrhea); 5 addressed quality of STI treatment; 5 the quality of family planning or reproductive health services; 4 malaria treatment; and the remaining studies addressed other communicable diseases (e.g. ARIs), or multiple diseases (e.g. "6 common illnesses").

Most interventions produced positive results for at least some outcome indicators. For instance, a study of the Ghanaian intervention to improve STI management at pharmacies, which evaluated outcomes using simulated clients, found that when offered treatment, 38% of simulated clients received appropriate oral medication at intervention pharmacies compared with 18% at control pharmacies. Counseling about partner notification was 40% in intervention pharmacies compared with 21% in control pharmacies, though no recommendation to use a condom was given at intervention pharmacies compared with 13% at control pharmacies [44]. Generally positive overall results were also observed for training programmes with non-pharmacy retailers. For example, the proportion of Nigerian patent medicine vendors recommending a correct drug dose for malaria increased from 9% in 2003 to 53% in 2004 [45], and in Kenya the proportion of antimalarial sales with adequate dosage increased from 32% in 1996 to 83% 3 months after training and to 90% 6 months after training [46].

Of the 26 interventions, data on average SES of the recipient population was provided for only one. Training of private practitioners in Pakistan clearly benefited a generally poor population, with an estimated median monthly household income of $48–72 and $48–61 in two communities [47]. General information on rural/urban settings was provided for 18 interventions and no information for 7. No studies provided any evidence on the distribution of benefits by relative SES.

### Franchising

Six interventions were identified, "Green Star" and "Green Key" in Pakistan, "Ray of Hope" in Ethiopia, "Janani" in Bihar State, India, "Sewa" in Nepal and "Top Reseau" in Madagascar. Evidence of impact on utilization or quality of health services was mixed.

Effectiveness of the Pakistan, Ethiopia and Bihar interventions was documented in a single study that used exit interviews to examine client satisfaction at franchised and non-franchised outlets [48]. Effectiveness of the "Green Star" and "Green Key" franchises implemented in Pakistan was jointly evaluated. Clients attending franchised private services were significantly more likely to report that they would return than those attending non-franchised services in Pakistan and significantly less likely in Ethiopia, with no statistically significant difference in Bihar. In all three settings there was no statistically significant difference between the franchise status of the clinic and perceptions of quality (that the service was better than others available) or in citing affordability as a preferred feature of the service [48].

The Nepali study examined client satisfaction with quality of care [49]. Clients at intervention clinics 'very satisfied' with cleanliness increased from 37% to 65%, and with the availability of essential equipment from 35% to 62% [49]. Clients were also reported to be more satisfied with the range of services offered in the intervention clinics (40% to 71%) and with privacy (38% to 72%) [49].

The Top Réseau study reported that coverage of modern contraceptives was higher for women with high exposure to the intervention (have accessed a franchised clinic and have been exposed to the IEC activities) than those with low or medium exposure [50].

Data on average SES of the recipient population was provided for one of the six interventions. The Nepali franchise network clearly benefited a generally poor population, with an estimated income per capita of $125 [49].

Evidence on the socioeconomic distribution of benefits within the recipient community was provided for the franchise networks in Pakistan, Ethiopia and Bihar State. The Green Star and Green Key interventions in urban Pakistan did not benefit relatively poor groups. Clients with income of $101– $250/month and with income greater than $251 were more likely to use franchised services than those earning less than $60/month. Clients with at least secondary schooling were also more likely to use franchised services compared to illiterate clients [48].

Evidence for the Ray of Hope intervention in Ethiopia was mixed. Clients with income of $101–$250/month were less likely to attend franchised services than those earning less than $60/month. However clients with primary education were more likely to attend franchised services compared to clients with the least education [48].

The evidence from Bihar was also mixed. There was no statistically significant association between attending franchised services and monthly household income [48]. Clients with no education were more likely to attend franchised services compared to clients with education [48].

Only general SES information was provided for the Top Réseau study.

### Accreditation

One intervention was identified, a network of accredited drug dispensing outlets (ADDO) implemented in rural and peri-urban Tanzania. The accreditation process, managed by the Tanzania Food Drug Authority (TFDA), aimed to improve access to affordable and quality medicines and pharmaceutical services through training and supervision of outlet dispensing staff, outlet inspections, marketing and public education [51]. The proportion of unregistered drugs decreased in both intervention and control areas, from 26% to 2% in the former, and from 29% to 10% in the latter [51].

Only general information on the rural and peri-urban status of the recipient populations was available, and no information was provided about ADDO customers' SES.

### Contracting-out

Three evaluated interventions were identified, of which one related to contracting-out hospital services in South Africa and two related to contracting-out primary health care services in South Africa and Lesotho.

Contracting-out of district hospitals to private-for-profit management was implemented in rural South Africa. The quality of care provided by three contracted hospitals was compared with that of three, paired public hospitals [52]. Public hospitals had better structural quality of care but contracted hospitals had better quality of nursing care in maternity and medical/surgical wards than public hospitals, similar nursing management quality, and overall, higher total nursing quality. No statistically significant differences in perinatal and maternal mortality rates were found between contracted and public hospitals [52].

General practitioners have for long been contracted on a part-time basis to provide primary care in rural towns in South Africa [53]. A quality of care study showed that patients with hypertension were less likely to have their blood pressure recorded when they sought care at contracted practices than at public health facilities.

Primary care services, drugs, laboratory tests and X-rays were provided in Lesotho to workers of a construction company and to local communities through a contract with a commercial medical company [53]. Overall, structural quality was similar between contracted and public providers. However, 37% of STI cases were treated correctly by contracted providers compared with 59% and 96% of cases treated in "large" and "small" public health facilities respectively.

Of the 3 evaluated interventions, data on the average SES of the recipient community was provided for 2. The individual GP and the company contract interventions clearly benefited generally poor users given that approximately 65% to 78% of them had an estimated household monthly income of less than $66 [53].

For the intervention to contract out district hospitals, rural location was the only information about SES provided.

## Discussion

This paper has reviewed through an "equity lens" [54] the results of a systematic literature review of the ways of working with for-profit providers of health services and public health commodities to improve the utilization and quality of essential health services, focusing specifically on the extent to which these interventions have been demonstrated to improve quality and utilization for poor and disadvantaged groups. This focus on whether the poor benefit is particularly important as programmes which work with private for-profit providers might be expected *a priori *to be pro-rich, since they generally require out-of-pocket payment (except in the case of a 100% value voucher). Available data indicate that poor people make significant use of the private sector, and that the quality of services they receive is at best variable. While a case can be made, therefore, for using public funds to work with for-profit providers, there is a need for much stronger evidence that such interventions can lead to health improvements for poor people.

The review confirms that international interest in working with the private sector has been translated into a significant number of innovative schemes. A total of 2483 references retrieved from our initial search of the published and grey literature related to one or more of 8 different areas of intervention (social marketing, vouchers, pre-packaging of drugs, franchising, accreditation, regulation, training and contracting-out). These interventions were implemented in a wide range of settings, and addressed a variety of different health problems.

Yet despite this large number of studies, relatively few qualified for inclusion as impact evaluations. In seeking to find evidence of impact we took a more liberal position than that of the EPOC group of the Cochrane Collaboration [55], and included any studies that had employed either a pre-post, controlled or pre-post with control design, with or without randomisation. We do not feel, therefore, that we have been overly rigid in our standards of "rigour" in evaluation design. However, even using these relatively loose criteria, it is clear that the ability of these interventions to produce a significant impact on quality and utilization of care is far from fully demonstrated.

Even fewer studies (5 interventions in total) reported data on the average SES of populations served, and therefore, of the beneficiaries of any improvements in quality or utilisation of care. However, although detailed data were lacking in the majority of studies, it is evident that many interventions have worked successfully in poor communities. For example, even where specific information about SES is not provided, rural districts in low-income countries are very likely to contain populations defined as absolutely poor by any standards and successful interventions in these contexts are likely to produce equity improvements.

We deliberately sought information about the socioeconomic distribution of benefits across socioeconomic groups and found information available for only 5 interventions. Strong evidence of benefits favouring the poorest was provided by the two ITN interventions (social marketing of ITNs in Tanzania and vouchers in Zambia), both of which used the private retail sector for distribution. In the case of Zambia, full value vouchers were provided so no out-of-pocket payment was required. In the Tanzanian programme, significantly pro-poor results for ITN coverage were achieved even though users were paying near-market prices for ITNs. Both these interventions operated at a relatively small scale and more evidence is needed of effectiveness at scale. Information about relative equity improvements was provided by the three franchising interventions, for which the socioeconomic distribution of impact was more mixed, with positive equity effects shown in some but not all programmes. This is difficult to explain without knowing more about the specific features of the interventions and study contexts. However, we can speculate that by targeting formally trained for-profit providers, the potential impact on the poorest is lessened and that positive equity effects might require targeting those providers predominantly used by poor people. Formative research is therefore needed to identify which providers are used by poor people and why, focusing on various dimensions of accessibility (geographic, social and financial). Where financial accessibility is a critical constraint, an assessment of alternative subsidy mechanisms should be considered (e.g. cash transfers, vouchers, provider subsidies).

The review identified a number of limitations of the literature, some of which have been alluded to already. First, there are few evaluations of impact that allow robust conclusions to be drawn. This is not surprising as many interventions were not set up as research exercises, and evaluation therefore had to fit around other implementation priorities. We do not wish to imply that other types of study are not of value, especially those which focus on description and analysis of implementation process. However, better evidence of impact is needed [56]. Other weaknesses of study design included limited justification for selection of control groups and few attempts to identify and control for potential confounders. Second, very few studies presented information about the SES of the target population or of programme beneficiaries. Among those studies included in the review, varied SES measures were used, hampering comparison of results across contexts. Finally, many studies assess relatively short-term effects under controlled conditions, leaving the long term impact and sustainability of the interventions open to question.

Finally our review had limitations. First, we searched only English and French language literature, and used search engines and databases that primarily index English language sources; for grey literature we restricted our search to sources that were web-accessible. Secondly, we encountered some difficulties in classifying interventions since many employ a mix of approaches, for instance social marketing and pre-packaging of drugs, or vouchers and training providers. We sought to classify studies by primary or main intervention. However, it should be noted that interventions included under the same sub-heading are in reality highly heterogeneous. Moreover, with complex interventions such as these, the context is likely to have a strong influence on both the manner of implementation and the intervention's success, meaning that any generalisation should be undertaken cautiously [11]. Finally, our conception of poverty is limited. We focused primarily on SES, but there are other dimensions of disadvantage that might be important, such as gender, ethnicity, and epidemiological vulnerability.

These limitations notwithstanding, practical suggestions for future work can be identified:

• The general SES and other features of the recipient community must be reported in order to assess whether results can be generalized across contexts.

• The socioeconomic distribution of programme benefits should be examined whenever possible. Measuring SES is challenging: income or expenditure are difficult to measure accurately, and may not capture the main dimensions of vulnerability; proxies such as asset indices are easier to collect but may raise other methodological and empirical difficulties [57]. Moreover, the benefits of assessing SES must be weighed against the feasibility and costs of collecting this information, especially where this involves large household surveys. Where household survey data on asset ownership are available from other sources, data on the same assets can be collected at exit interviews with specific private providers, and clients located in the overall SES distribution by applying asset weights from the household survey [14,58].

• Information on relative SES must be interpreted with care, and in light of the characteristics of the overall study population: in a relatively homogeneous, poor setting, beneficiaries who appear relatively better off may still be poor in absolute terms compared with the national distribution of income.

• An outcome that favours the poor does not on its own imply such interventions are good value for money; investments in improving quality of care in the private sector need to be compared with the return from investment in the public sector, including the ability of such investments to switch use away from low quality private care to better quality public services.

In conclusion, it is not possible to prove from the available literature that private sector interventions benefit the poor and improve equity. However, the fact that many such interventions have operated successfully in relatively poor regions or poor countries indicates that it is possible the poor do benefit significantly – the challenge for the future is to design evaluations and report results in ways that can assess this clearly, and indicate how equity can be enhanced.

## Competing interests

The author(s) declare that they have no competing interests.

## Authors' contributions

CG, KH and AM defined the scope of the research subject

EP developed the search strategy, undertook the search, reviewed the literature and summarised the search findings

KH, CG and EP drafted the manuscript

AM, CG, and KH critically revised the manuscript

All authors approved the final manuscript

## Supplementary Material

Additional file 1 Summary of evaluated interventions. The file contains information about the type, location and date of each intervention, the design of their evaluation study and the outcomes and socioeconomic measures used. Click here for file
