# Supplementary material for: Can working with the private for-profit sector improve utilization of quality health services by the poor? A systematic review of the literature
Source: Int J Equity Health. 2007 Nov 7;6:17. doi: 10.1186/1475-9276-6-17 (PMC2186328; doi:10.1186/1475-9276-6-17)
Supplement: Additional file 1 — Summary of evaluated interventions. The file contains information about the type, location and date of each intervention, the design of their evaluation study and the outcomes and socioeconomic measures used. [file 1475-9276-6-17-S1.doc]

## Additional File 1. Summary of evaluated interventions

| **Intervention evaluated (reference)** | **Location and date intervention initiated** | **Description of Intervention** | **Utilisation and quality outcome measures** | **Study design** | **Average SES measure** | **Relative SES measure** |
| --- | --- | --- | --- | --- | --- | --- |
| **1. Social marketing (SM), vouchers and pre-packaging** | | | | | | |
| My Future First [1-3] | Towns of Conakry and Kankan, Guinea, 1997 | SM of condoms and other contraceptives through retail outlets, hotels, bars, pharmacies, health centres, peer educators | Likelihood ratio of contraceptive use in the population exposed to the intervention to that of the non-exposed | Pre-post with control | Urban areas |  |
| Tsa Banana [1, 3, 4] | Lobatse, Botswana, 1995 | SM of condoms through peer educators, retail outlets, magazine inserts. | Likelihood ratio of contraceptive use in the population exposed to the intervention to that of the non-exposed | Pre-post with control | Urban area |  |
| 100% Jeune [5-7] | Towns of Yaoundé and Douala, Cameroon, 2000 | SM of condoms through retail outlets | Proportion of adolescents who reported condom use | Pre-post with control | Urban areas |  |
| Horizon Jeunes [8] [1] [9] | Edea, Cameroon, 1996 | SM of condoms and other contraceptives through peer educators, retail outlets, pharmacies and youth clubs | Proportion of adolescents who reported use of contraceptive methods | Pre-post with control | Urban area |  |
| Soweto adolescent reproductive health programme [10] [1] | Soweto and Umlazi towns, South Africa, 1994 | SM of condoms through retail outlets and peer educators | Proportion of adolescents who reported use of contraceptive methods | Pre-post with control | Urban areas |  |
| Salama [11] | Tanzania, December 1993 | SM of condoms through NGOs, pharmacies, retail outlets, bars, gas stations | Proportion of adolescents who reported condom use | Pre-post |  |  |
| Social marketing of oral rehydration therapy [12] | Rural district of Kakamega, Western Kenya, 1986 | SM of ORT through retail outlets | Proportion of episodes of acute diarrhoea in children under five years treated with ORS | Controlled | Rural district |  |
| Social marketing of iron and folic-acid supplements [13] [14] | Calasiao, Binmaley and Santa Barbara, Pangasinan areas, Northern Luzon, Philippines, November 1998 | SM of iron and folic acid supplements for women of reproductive age through drug outlets, village health workers, public health facilities and school teachers | Proportion of women who took weekly iron-folic acid supplements | Pre-post | Rural areas |  |
| KINET [15] | Kilombero and Ulanga districts, Southern Tanzania, 1997 | SM of ITNs through retail outlets. Subsidies to pregnant women and mothers of young children through vouchers from public clinics. | Proportion of children with a net | Pre-post | Rural districts  Median monthly household expenditure $77-96, 1997 [16] | Household income |
| KINET [17] | As above | As above | Proportion of children under five years who slept under an ITN the previous night; number of child deaths prevented | Pre-post (coverage outcomes); controlled (mortality outcomes) | As above |  |
| KINET [18] | As above | As above | Proportion of women who used a voucher to buy an ITN | Pre-post | As above | Household income/ education/ asset quintiles |
| KINET [19] | As above | As above | Proportion of households with net | Pre-post | As above | Household asset quintiles |
| KINET [20] | As above | As above | Proportion of households with net | Pre-post with control | As above | Household income quartiles |
| Prepackaged drugs for childhood febrile illnesses [21] | Idere town and surrounding areas, Oyo State Ukehe community, Igbo Enugu State Mbaugwu community, Abia State, Nigeria, 1999 | SM of prepackaged chloroquine and cotrimoxazole distributed through patent medicine vendors, village health workers, clinics. | Proportion of children with fever who received antimalarials; proportion of those with ARI who received antibiotics | Pre-post | Urban areas |  |
| Clear Seven [22] | Jinja, Luweero and Mbarara rural districts and 2 divisions of Kampala, Uganda, 1999 | SM of pre-packaged treatment kits for STIs through retail outlets | Proportion of men with urethral discharge who complied to the treatment regimen; proportion of those who used condoms during treatment; proportion of those who were cured | Controlled | Combination of rural districts and urban divisions |  |
| Vouchers for ITN distributed during measles vaccination campaign [23] | Urban district of Kalulushi, Zambia, 2003 | Distribution of vouchers for ITNs redeemed through retail outlets | Proportion of children who slept under an ITN the previous night; proportion of households with at least one ITN | Pre-post | Urban district | Household asset quintiles |
| Vouchers for sexual and reproductive health care [24, 25] | Managua, Nicaragua, 2000 | Distribution of vouchers for free care redeemed at NGO, public, private clinics. | Likelihood ratio of heath services use in voucher-receivers to that of non-receivers ; likelihood ratio of contraceptive use in voucher-receivers to that of non-receivers | Controlled | Urban area |  |
| Vouchers for sexually transmitted infections (STI) care [26] | Managua, Nicaragua, 1995 | Distribution of vouchers for free STI testing and treatment redeemed at private and NGO clinics. | Evolution of HIV prevalence rate in sex workers | Controlled | Urban area |  |
| **2. Regulation** |  |  |  |  |  |  |
| Pharmaceutical ban [27] | Districts of Kathmandu, Lalitpur and Bhaktapur, Kathmandu Valley, Nepal, 1997 | Prohibition to export, import, produce, transport, store, sale and distribute Analgin and its combination products | Proportion of retail outlets with analgin or its combination products | Pre-post |  |  |
| Regulation of  pharmacy practice  [28] | Savannakhet province, Lao P.D.R, 1988 | Intensive supervision of quality of services of private pharmacies | Scores in quality of drug storage, presence of drug advertisement and availability of essential dispensing materials; proportion of customers who received drug information and appropriately packed and labelled drugs | Randomized controlled study |  |  |
| **3.Training** |  |  |  |  |  |  |
| Training of private doctors on IMCI implementation [29] | Rural villages in Dumka and Bettiah districts, Bihar State, India, no date | Training on IMCI algorithm through INFECTOM (INformation, performance FEedback, ConTracting, Ongoing Monitoring) | Proportion of cases appropriately managed | Pre-post | Rural areas |  |
| Training of private doctors on IMCI implementation [30] | Sultanabad and Rehri Goth communities, Karachi area, Pakistan, 1997 | As above | Proportion of cases appropriately managed; average number of key practices that ill children with any symptoms received | Pre-post | Two areas with median monthly household income of $48-$72 and $48-$61 |  |
| Training of private doctors on management of ARI and diarrhoea in children [31] | Five areas of Tlaxcala State, Mexico, 1993 | In-service training through 5-day course on diarrhoea and ARI | Proportion of providers who correctly managed diarrhoea / ARI; proportion of providers who provided adequate counselling | Pre-post |  |  |
| Training of private doctors on family planning (FP) services [32] | Northern India, 1992 | Two half-day sessions and 1 half-day follow up session | Proportion of practitioners who recommended oral contraceptive pills | Pre-post |  |  |
| Training of private midwives in STI management and post-abortion care services [33] | Three districts, Eastern region of Ghana, 1996 | Initial and refresher training sessions | Number of clients provided with STI and post-abortion care; proportion of facilities where clinical sessions and new equipment were added; proportion of patients who perceived services had improved | Controlled |  |  |
| Training of private midwives in reproductive health youth services and client provider interaction [34] | Ashanti, Ahafo and Eastern regions of Ghana, 1999 | Five-month self-directed training including practical exercises, group peer review and facility visits | Proportion of midwives who performed critical counselling tasks; proportion of clients who reported being very satisfied or satisfied | Pre-post with control | Urban and rural areas |  |
| Training of pharmacy workers in STI management [35] | Lima, Peru, 1995 | Training on management and prevention of male urethritis and genital ulcer, vaginal discharge and pelvic inflammations through 8-hour course or 1.5 to 2 hour on site session | Proportion of STI cases managed in accordance with training guidelines | Randomised controlled study, with pre-post data | Urban area |  |
| Training of pharmacy workers in STI management[36] | As above | As above through 3-month pilot of four-90 minute luncheon seminars and 2-month implementation phase of three- 90 minute luncheon seminars in six other divisions | As above | Randomised controlled study | Low income urban areas |  |
| Training of pharmacy workers in STI management [37] | Accra, Ghana, 1997 | Training in management of urethral discharge, gonorrhoea and chlamydia through one day training course in history taking, examination, treatment, condom promotion, partner notification, health education, counselling and STI record keeping | Proportion of STI cases who were adequately managed | Controlled | Urban area |  |
| Training of pharmacy workers in STI management [38] | Transportation routes from Naubise to Birgunj and Janakpur, Central region of Nepal, 1995 | Training on STI drug dispensing practices, HIV and STI prevention, condom promotion | Proportion of pharmacists who recommended an injection; proportion of those who dispensed adequate drugs and dosages; proportion of those who suggested condom use and sold condoms | Pre-post |  |  |
| Training of pharmacy workers in management of ARI, STI and dispensing practices [39-42] | Hanoi, Vietnam,  1998 | Multi-component intervention including training on dispensing practices for ARI, STI, steroids and antibiotic drugs, through two 45-minute face-to-face training sessions, regulation enforcement, peer monitoring | Proportion of ARI / STI cases correctly managed; proportion of antibiotics / steroids dispensed with prescriptions | Randomised controlled study with pre-post data | Urban area |  |
| Training of pharmacy workers in management of ARI, STI and dispensing practices [40] | Two districts of Bangkok, 1998 | As above with 2-day group seminar | Proportion of ARI / STI cases correctly managed; proportion of antibiotics dispensed in adequate dosage; proportion of steroids dispensed with prescriptions | Randomised controlled study with pre-post data | Urban areas |  |
| Training of pharmacy workers in diarrhoea management [43] | Six towns including Nairobi, Kenya  no date | Training on knowledge, drug sales and patient communication for diarrhoea management. Face-to-face meetings, 2-3 hour group training (pilot) | Proportion of cases who received ORS or anti-diarrhoeal drugs | Pre-post | Urban areas |  |
| Training of pharmacy workers in diarrhoea management [44] | Urban towns of Nairobi, Nakuru, Kisumu, Kenya, no date | As above (intervention) | As above | Pre-post with control | As above |  |
| Training of pharmacy workers in diarrhoea management [44] | Towns of Jakarta and Bogor, Tangerang and Bekasi area, Indonesia, no date | As above with 2-day group training | Proportion of cases who received ORS or anti-diarrhoeal drugs | Randomised controlled study | Urban areas |  |
| Training of pharmacy workers on family planning services [45] | Cities of Cotonou and Porto Novo, Southern Benin,  1998-1999 | Training through one half-day workshop, home study | Practice scores in counselling new users and prescribing contraceptive | Controlled | Urban areas |  |
| Training of pharmacy workers on family planning services [46] | As above, 2001 | Two 1-day training sessions to follow up above intervention | Proportion of workers achieving or surpassing the acceptable performance level | As above | As above |  |
| Training of drug retailers in management of childhood fever and malaria [47] | Chonyi area, district of Kilifi, Kenya, 1996 | 3-day training workshops, follow up visits to shops, 2-day refresher session, and provision of job aids (pilot) | Proportion of drug sales for children with fever which included an antimalarial or antipyretic drugs; proportion of antimalarial sales where an adequate dose of chloroquine was bought; proportion of cases for which an adequate dose of chloroquine was administered | Pre post | Rural district |  |
| Training of drug retailers in management of childhood fever and malaria [48] | District of Kilifi, Kenya, 1999 | Four-day workshops, follow up visits to shops, 1-day refresher session, provision of job aids and community mobilisation (intervention) | Proportion of OTC anti-malarial drug users receiving adequate drug dosage | Pre-post with control | As above |  |
| Training of drug retailers on OTC antimalarial selling practices [49] | Kenya, no date | Training on OTC anti-malarial dispensing practices through outlet visits, distribution of IEC material | Proportion of households who received the recommended first line antimalarial drug; proportion of those who used the correct dose | Pre post | Rural district |  |
| Training of drug retailers on emergency contraception (EC) [50] | Hat Yai, Southern Thailand, 2000 | Training through one-day session including a lecture, dispensing practices, role play activities | Number of retailers who correctly dispensed and advised on EC | Randomised controlled study with pre post data | Urban area |  |
| “Vendor to Vendor” training on antimalarial selling practices [51] | Bungoma district, Western Kenya, 2000 | Distribution of job aids and client posters to shops, kiosks, pharmacies and private clinics | Proportion of malaria cases who received the recommended first-line anti-malarial; proportion of those who received correct drug dosage; proportion of outlets where adequate drugs were available | Controlled |  |  |
| Training of Patent Medicine Vendors on management of malaria in children [52] | Aba South and Aba North areas, southern part of Aba State, Southeastern Nigeria, 2003 | One-day training on management of childhood fevers, including drug delivery, referral and ITN usage. Distribution of job aids | Proportion of providers who correctly managed malaria; proportion of caregivers who received correct drug dosage | Pre post |  |  |
| Training of drug retailers on antibiotics selling practices [53] | Three rural villages, Northern Philippines, 1996 | Informal half-day focus group discussions and outlet visits | Proportion of retailers who sold antibiotics without requesting prescriptions | Randomised controlled study with pre post data | Rural areas |  |
| Training of private doctors and drug retailers on IMCI [52] [54] | Four sub-counties of Kinyogoga, Kamira, Makulobita and Ngoma, Luwero area, eastern central region of Uganda, 2003 | Negotiation sessions and visits to providers for behaviour change in management of ARI, diarrhoea and malaria in children | Proportion of providers who engaged in key child survival practices | Pre post | Rural areas |  |
| Training of private doctors, pharmacy workers and patent medicine vendors on STI management [55] [9] | Western section of Benin City, Edo State, Nigeria, 1997 | Training on STI diagnosis and treatment, including 30 hours of lectures, demonstrations, practical exercises | Proportion of adolescents who reported condom use | Randomised controlled study with pre post data | Urban area |  |
| Training of private doctors and pharmacists in sexual and reproductive health youth services [9] | Nyeri, Kenya, 1998 | Training on making sexual health services youth-friendly | Proportion of adolescents who reported condom use | Pre post with control | Urban and rural areas |  |
| Training of private doctors and paramedics [56] | Five towns, Pakistan, 2000 | Refresher training for doctors and paramedics who were members of the Key Social Marketing (KSM) project | Proportion of providers who gave full and accurate instructions of how to take the pill; proportion of those who carried out a blood pressure check | Pre post | Urban areas |  |
| Training of pharmacy workers and drug retailers on diarrhoea management [57] | Three subdistricts, northern Bangkok Thailand, 1991 | Three hour session for pharmacists, telephone call to ‘partially trained’ pharmacists and drug sellers, distribution of educational material | Proportion of providers who prescribed ORS, antibiotics or antidiarrhoeal drugs in the treatment of watery diarrhoea and dysentery | Randomised controlled study with pre post data | Urban areas |  |
| Training of pharmacy workers and drug retailers on management of ARI [58] | Kampala district, Uganda, 2000 | Three morning face-to-face sessions, distribution of educational materials | Proportion of providers who asked specific questions to assess childhood conditions; average number of questions asked by providers; proportion of providers who dispensed the recommended first-line antibiotic; proportion of providers who referred acute and severe ARI | Pre post with control |  |  |
| **4. Franchising** |  |  |  |  |  |  |
| Green Star/Green Key [59] | Urban Pakistan, 1997 | Network of pharmacies, clinics, hospitals, physicians, paramedics trained on reproductive health (RH) and family planning (FP) services | Proportion of clients who reported that they would return, that services were better than others available or who cited affordability a preferred feature of the service | Controlled | Urban areas | Monthly household income  Level of education attained |
| Ray of Hope [59] | Addis Ababa, Oromia and Amhara regions, Ethiopia, 2000 | Networks of clinics, community health agents, birth attendants, marketplace providers trained in FP, STDs, HIV/AIDS services counselling and referral procedures | Proportion of clients who reported that they would return, that services were better than others available or who cited affordability a preferred feature of the service | Controlled | Urban areas | Monthly household income  Level of education attained |
| Janani [59] | Bihar State, India, 1997 | Network of providers for RH, FP and STDs | Proportion of clients who reported that they would return, that services were better than others available or who cited affordability a preferred feature of the service | Controlled | Urban and rural areas | Monthly household income  Level of education attained |
| Sewa [60] | Rupandehi district, Nepal, 2002 | Network of nurses and paramedics for RH and FP | Proportion of clients who reported being “very satisfied” with different elements of service quality | Pre post with control | District with annual per capita income of $125 |  |
| Top Réseau [61] | Taomisina province, Madagascar, 2001 | Network of clinics for STI diagnosis, FP and RH counselling services and IEC activities | Proportion of clients with low, medium or high level of programme exposure who reported use of contraceptive methods | Pre post with control | Urban and rural areas |  |
| **5.Accreditation** |  |  |  |  |  |  |
| ADDOs [62] | Ruvuma region, Tanzania, 2003 | Training and supervision of outlet staff, outlet inspections, marketing and public education | Proportion of unregistered drugs available | Pre post with control | Rural and peri-urban regions |  |
| **6. Contracting Out** |  |  |  |  |  |  |
| Contracting out for hospital care [63] | South Africa, 1995 | Contracts with management company to run 3 rural district hospitals | Proportion of “maximum possible scores” obtained in structural quality and quality of nursing care; mean perinatal and maternal mortality rates | Controlled | Rural district |  |
| Individual GP contract [64] | Western and Eastern Cape, South Africa, no date | Provinces contract private GPs part-time for primary care services in rural towns | Proportion of STI cases who are diagnosed or treated correctly; Scores in structural quality (adequate emergency equipment, range of services offered, appropriate health education material on display, supply of essential drugs); Proportion of diabetes / hypertension cases for which key practices are performed | Controlled | Low income groups 65% to 78% of users from households with monthly income < $US 66  Rural towns |  |
| Company contract  [64] | Lesotho, no date | Clinics contracted to provide primary care to workers of parastatal company and local communities | Proportion of STI cases who are diagnosed or treated correctly; Scores in structural quality (adequate emergency equipment, range of services offered, appropriate health education material on display, supply of essential drugs); Proportion of diabetes / hypertension cases for which key practices are performed | Controlled | Remote rural areas  Two sites, of which one provides care to users with monthly household income < $US 66 |  |

# References

1. Agha S: **A quasi-experimental study to assess the impact of four adolescent sexual health interventions in sub-Saharan Africa**. *Int Fam Plan Perspect* 2002, **28**(2):67-118.

2. Van Rossem R, Meekers D: *An evaluation of the effectiveness of targeted social marketing to promote adolescent reproductive health in Guinea*. Washington, DC: PSI; 1999.

3. Agha S: *An evaluation of adolescent sexual health programs in Cameroon, Botswana, South Africa and Guinea*. Washington, DC: PSI; 2000.

4. Meekers D, Stallworthy G, Harris J: *Changing adolescents' beliefs about protective sexual behavior: The Bostwana Tsa Banana Program*. Washington, DC: PSI; 1997.

5. Meekers D, Agha S, Klein M: *The impact on condom use of the "100% Jeune" social marketing program in Cameroon*. Washington, DC: PSI; 2003.

6. Meekers D, Agha S, Klein M: **The impact on condom use of the "100% Jeune" social marketing program in Cameroon**. *J Adolesc Health* 2005, **36**(6):530.

7. Plautz A, Meekers D: *Evaluation of the Reach and Impact of the "100% Jeune" Youth Social Marketing Program in Cameroon*. Washington, DC: PSI; 2003.

8. Van Rossem R, Meekers D: **An evaluation of the effectiveness of targeted social marketing to promote adolescent and young adult reproductive health in Cameroon**. *AIDS Educ Prev* 2000, **12**(5):383-404.

9. Alford S, Cheetham N, Hauser D: *Science and success in developing countries: holistic programs that work to prevent teen pregnancy, HIV and sexually transmitted infections*. Washington, DC: Advocates For Youth; 2005.

10. Meekers D: *The effectiveness of targeted social marketing to promote adolescents' reproductive health: the case of Soweto, South Africa*. Washington, DC: PSI; 1998.

11. Eloundou-Enyegue P, Meekers D, Calves A: *From awareness to adoption: the effect of AIDS education and condom social marketing on condom use in Tanzania (1993-1996)*. Washington, DC: PSI; 1998.

12. Kenya PR, Gatiti S, Muthami LN, Agwanda R, Mwenesi HA, Katsivo MN, Omondi O, Surrow A, Juma R, Ellison RH *et al*: **Oral rehydration therapy and social marketing in rural Kenya**. *Soc Sci Med* 1990, **31**(9):979-987.

13. Paulino LS, Angeles-Agdeppa I, Etorma UM, Ramos AC, Cavalli-Sforza T: **Weekly iron-folic acid supplementation to improve iron status and prevent pregnancy anemia in Filipino women of reproductive age: the Philippine experience through government and private partnership**. *Nutr Rev* 2005, **63**:109-115.

14. Angeles-Agdeppa I, Paulino LS, Ramos AC, Etorma UM, Cavalli-Sforza T, Milani S: **Government-Industry Partnership in Weekly Iron-Folic Acid Supplementation for Women of Reproductive Age in the Philippines: Impact on Iron Status**. *Nutr Rev* 2005, **2**:116-125.

15. Abdulla S, Schellenberg JA, Nathan R, Mukasa O, Marchant T, Smith T, Tanner M, Lengeler C: **Impact on malaria morbidity of a programme supplying insecticide treated nets in children aged under 2 years in Tanzania: community cross sectional study**. *BMJ* 2001, **322**:270-273.

16. Schellenberg JA, Mukasa O, Abdulla S, Marchant T, Lengeler C, Kikumbih N, Mshinda H, Nathan R: **Ifakara DSS, Tanzania**. In: *Population and health in developing countries.* Edited by INDEPTH Network. Ottawa: International Development Research Centre; 2001.

17. Schellenberg JR, Abdulla S, Nathan R, Mukasa O, Marchant TJ, Kikumbih N, Mushi AK, Mponda H, Minja H, Mshinda H *et al*: **Effect of large-scale social marketing of insecticide-treated nets on child survival in rural Tanzania**. *Lancet* 2001, **357**:1241-1247.

18. Mushi AK, Schellenberg JR, Mponda H, Lengeler C: **Targeted subsidy for malaria control with treated nets using a discount voucher system in Tanzania**. *Health Policy Plan* 2003, **18**(2):163-171.

19. Nathan R, Masanja H, Mshinda H, Schellenberg JA, de Savigny D, Lengeler C, Tanner M, Victora CG: **Mosquito nets and the poor: can social marketing redress inequities in access?** *Trop Med Int Health* 2004, **9**(10):1121-1126.

20. Kikumbih N, Hanson K, Mills A, Mponda H, Schellenberg JA: **The economics of social marketing: the case of mosquito nets in Tanzania**. *Soc Sci Med* 2005, **60**(2):369-381.

21. Brieger W, Salako L, Umeh R, Agomo P, Afolabi B, Adeneye A: **Promoting pre-packaged drugs for prompt and appropriate treatment of febrile illnesses in rural Nigerian communities**. *Int Q Community Health Educ* 2002-2003, **21**(1):19-40.

22. Jacobs B, Kambugu FS, Whitworth JA, Ochwo M, Pool R, Lwanga A, Tifft S, Lule J, Cutler JR: **Social marketing of pre-packaged treatment for men with urethral discharge (Clear Seven) in Uganda**. *Int J STD AIDS* 2003, **14**(3):216-221.

23. Grabowsky M, Farrell N, Hawley W, Chimumbwa J, Hoyer S, Wolkon A, Selanikio J: **Integrating insecticide-treated bednets into a measles vaccination campaign achieves high, rapid and equitable coverage with direct and voucher-based methods**. *Trop Med Int Health* 2005, **10**(11):1151-1160.

24. Meuwissen LE, Gorter AC, Knottnerus JA: **Perceived quality of reproductive care for girls in a competitive voucher programme. A quasi-experimental intervention study, Managua, Nicaragua**. *Int J Qual Health Care* 2006, **18**(1):35-42.

25. Meuwissen LE, Gorter AC, Knottnerus AJ: **Impact of accessible sexual and reproductive health care on poor and underserved adolescents in Managua, Nicaragua: a quasi-experimental intervention study**. *J Adolesc Health* 2006, **38**(1):56.

26. Sandiford P, Gorter A, Salvetto M: **Vouchers for health: using voucher schemes for output-based aid**. *Public Policy for the Private Sector, an online journal of the World Bank* 2002.

27. Dange Chettri G, Kafle K, Karkee S, Rajubhandari V, Humagain B: **Effect of regulatory intervention on drug availability in Nepal**. In: *Proceedings of International Conferences on Improving Use of Medicines (ICIUM): 2004 March 30-April 2; Chiang Mai*.

28. Stenson B, Syhakhang L, Lundborg CS, Eriksson B, Tomson G: **Private pharmacy practice and regulation. A randomized trial in Lao P.D.R**. *Int J Technol Assess Health Care* 2001, **17**(4):579-589.

29. Chakraborty S, D'Souza SA, Northrup RS: **Improving private practitioner care of sick children: testing new approaches in rural Bihar**. *Health Policy Plan* 2000, **15**(4):400-407.

30. Luby S, Zaidi N, Rehman S, Northrup R: **Improving private practitioner sick-child case management in two urban communities in Pakistan**. *Trop Med Int Health* 2002, **7**(3):210-219.

31. Bojalil R, Guiscafre H, Espinosa P, Viniegra L, Martinez H, Palafox M, Gutierrez G: **A clinical training unit for diarrhoea and acute respiratory infections: an intervention for primary health care physicians in Mexico**. *Bull World Health Organ* 1999, **77**(11):936-945.

32. Barge S, Khan I, Patel B, Khan M: *Use of private practitioners for promoting oral contraceptive pills in Gujarat*. New Delhi: Centre for Operations Research and Training and the Population Council; 1995.

33. Fullerton J, Johal K, Fort A: *Study of the effects of incorporating selected reproductive health services on family planning services: a case study in the Eastern region of Ghana*. Chapel Hill: Intrah; 1999.

34. Newman C, Ambegaokar M, Abbey M, Muhawenimana A, Combary P: *Evaluation of the GRMA/PRIME self-directed learning, client provider interaction and adolescent reproductive health initiative*. Chapel Hill: Intrah; 2001.

35. Garcia PJ, Gotuzzo E, Hughes JP, Holmes KK: **Syndromic management of STDs in pharmacies: evaluation and randomised intervention trial**. *Sex Transm Infect* 1998, **74 Suppl 1**:153-158.

36. Garcia P, Hughes J, Carcamo C, Holmes KK: **Training pharmacy workers in recognition, management, and prevention of STDs: district-randomized controlled trial**. *Bull World Health Organ* 2003, **81**(11):806-814.

37. Adu-Sarkodie Y, Steiner MJ, Attafuah J, Tweedy K: **Syndromic management of urethral discharge in Ghanaian pharmacies**. *Sex Transm Infect* 2000, **76**(6):439-442.

38. Tuladhar SM, Mills S, Acharya S, Pradhan M, Pollock J, Dallabetta G: **The role of pharmacists in HIV/STD prevention: evaluation of an STD syndromic management intervention in Nepal**. *AIDS* 1998, **12 Suppl 2**:81-87.

39. Chalker J, Chuc NT, Falkenberg T, Tomson G: **Private pharmacies in Hanoi, Vietnam: a randomized trial of a 2-year multi-component intervention on knowledge and stated practice regarding ARI, STD and antibiotic/steroid requests**. *Trop Med Int Health* 2002, **7**(9):803-810.

40. Chalker J, Ratanawijitrasin S, Chuc NT, Petzold M, Tomson G: **Effectiveness of a multi-component intervention on dispensing practices at private pharmacies in Vietnam and Thailand-a randomized controlled trial**. *Soc Sci Med* 2005, **60**(1):131-141.

41. Chuc NT, Larsson M, Do NT, Diwan VK, Tomson GB, Falkenberg T: **Improving private pharmacy practice: a multi-intervention experiment in Hanoi, Vietnam**. *J Clin Epidemiol* 2002, **55**(11):1148-1155.

42. Larsson M: **Antibiotic use and resistance: assessing and improving utilisation and provision of antibiotics and other drugs in Vietnam**. *PhD thesis.* Karolinska Institutet; 2003.

43. Goel P, Makhulo J, Mwangi G: **Working with pharmacists in Kenya**. *Dialogue on Diarrhoea* 1994(55).

44. Ross-Degnan D, Soumerai SB, Goel PK, Bates J, Makhulo J, Dondi N, Sutoto, Adi D, Ferraz-Tabor L, Hogan R: **The impact of face-to-face educational outreach on diarrhoea treatment in pharmacies**. *Health Policy Plan* 1996, **11**(3):308-318.

45. Ambegaokar M, Capo-Chichi V, Sebikali B, Echitey N: *Assessing the performance of pharmacy agents in counselling family planning users and providing the pill in Benin: an evaluation of Intrah/PRIME and PSI training assistance to the Benin social marketing program*. Chapel Hill: Intrah; 2003.

46. Combary P, Onanga B, Houeto D, Capo-Chichi V, Houendehoto Z, Capo-Chichi S: *Follow up of performance by pharmacy agents trained in contraceptive technology between 1998 and 2001 in Benin Technical report 35*. Chapel Hill: Intrah; 2003.

47. Marsh VM, Mutemi WM, Muturi J, Haaland A, Watkins WM, Otieno G, Marsh K: **Changing home treatment of childhood fevers by training shop keepers in rural Kenya**. *Trop Med Int Health* 1999, **4**(5):383-389.

48. Marsh VM, Mutemi WM, Willetts A, Bayah K, Were S, Ross A, Marsh K: **Improving malaria home treatment by training drug retailers in rural Kenya**. *Trop Med Int Health* 2004, **9**(4):451-460.

49. Muturi J: **Lessons learnt in training retail sellers on correct use of OTC anti-malaria drugs in Kenya**. In: *Interventions to Improve the Role of Medicine Sellers in Malaria Case Management for Children in Africa.* Edited by Brieger WR, Unwin A, Green M, Meek S. Arlington: Malaria Consortium and BASICS; 2005.

50. Ratanajamit C, Chongsuvivatwong V, Geater AF: **A randomized controlled educational intervention on emergency contraception among drugstore personnel in southern Thailand**. *J Am Med Women Assoc* 2002, **57**(4):196-199.

51. Tavrow P, Shabahang J, Makama S: **Vendor-to-vendor education to improve malaria treatment by private drug outlets in Bungoma District, Kenya**. *Malar J* 2003, **2**:10.

52. Greer G, Akinpelumi A, Madueke L, Plowman B, Fapohunda B, Tawfik Y, Holmes R, Owor J, Gilpin U, Clarence C *et al*: **Improving management of childhood malaria in Nigeria and Uganda by improving practices of patent medicine vendors**. In*.* Arlington: BASICS II and USAID; 2004.

53. Sia S, Valerin J: **The effect of an intervention on the drug-selling behaviour of sarisari storekeepers in some villages in the Philippines** In: *Proceedings of International Conferences on Improving Use of Medicines (ICIUM): 1997 April 1-4; Chiang Mai*.

54. Tawfik Y, Nsungwa-Sabitii J, Greer G, Owor J, Kesande R, Prysor-Jones S: **Negotiating improved case management of childhood illness with formal and informal private practitioners in Uganda**. *Trop Med Int Health* 2006, **11**(6):967-973.

55. Okonofua F, Coplan P, Collins S, Oransaye F, Ogunsakin D, Ogonor J, Kaufman J, Heggebhougen K: **Impact of an intervention to improve treatment-seeking behaviour and prevent sexually transmitted diseases among Nigerian youths**. *Int J Infect Dis* 2003, **7**:61-73.

56. Palmer L: *Private sector providers: do they behave the way they say they do?* Bath: Futures Group; 2003.

57. Podhipak A, Varavithya W, Punyaratabandhu P, Vathanophas K, Sangchai R: **Impact of an educational program on the treatment practices of diarrheal diseases among pharmacists and drugsellers**. *Southeast Asian J Trop Med Public Health* 1993, **24**(1):32-39.

58. Tumwikirize WA, Ekwaru PJ, Mohammed K, Ogwal-Okeng JW, Aupont O: **Impact of a face-to-face educational intervention on improving the management of acute respiratory infections in private pharmacies and drug shops in Uganda**. *East Afr Med J* 2004, **Suppl**:25-32.

59. Stephenson R, Tsui AO, Sulzbach S, Bardsley P, Bekele G, Giday T, Ahmed R, Gopalkrishnan G, Feyesitan B: **Franchising reproductive health services**. *Health Serv Res* 2004, **39**:2053-2080.

60. Agha S, Karim AM, Balal A, Sossler S: *A quasi-experimental study to assess the performance of a reproductive health franchise in Nepal*. Washington, DC: Commercial Market Strategies; 2003.

61. Plautz A, Meekers D, Neukom J: *The impact of the Madagascar TOP Réseau social marketing program on sexual behaviour and use of reproductive health services*. Washington, DC: PSI; 2003.

62. Sigonda-Ndomondo M, Kowero O, Alphonce E, Mbwasi R, Shirima R, Frankiewicz C, Taylor M, Heltzer N, Clark M: **Accredited Drug Dispensing Outlets: A Novel Public-Private Partnership**. In: *Proceedings of the conference on Strategies for Enhancing Access to Medicines (SEAM) December 2003; Dar es Salaam*: Management Sciences for Health; 2003.

63. Broomberg J, Masobe P, Mills A: **To purchase or to provide? The relative efficiency of contracting out versus direct public provision of hospital services in South Africa**. In: *Private health providers in developing countries:serving the public interest?* Edited by Bennett S, McPake B, Mills A. London: Zed Books; 1997.

64. Mills A, Palmer N, Gilson L, McIntyre D, Schneider H, Sinanovic E, Wadee H: **The performance of different models of primary care provision in Southern Africa**. *Soc Sci Med* 2004, **59**(5):931-943.
